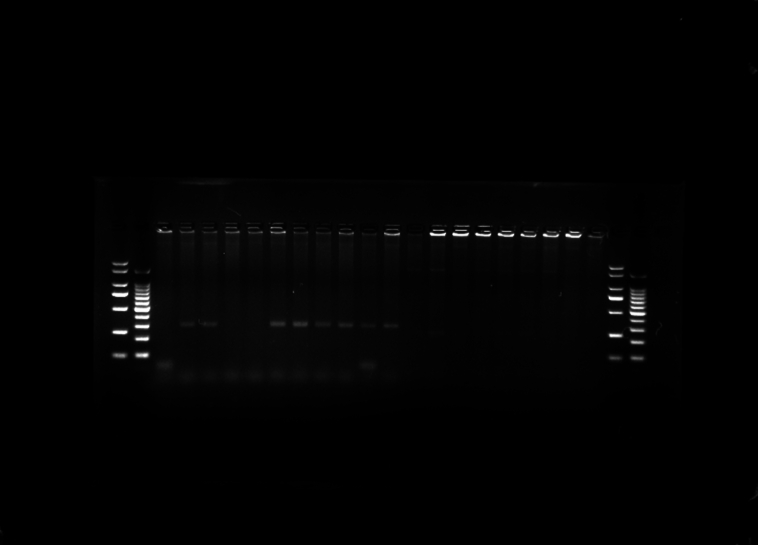# Functional study of the *ST6GAL2* gene regulating skeletal muscle growth and development

**DOI:** 10.1016/j.heliyon.2024.e37311

**Published:** 2024-09-01

**Authors:** Tao Wang, Bo Ran, Yingyu Luo, Jideng Ma, Jing Li, Penghao Li, Mingzhou Li, Diyan Li

**Affiliations:** a School of Pharmacy, Chengdu University, Chengdu, 610106, China; b Sichuan Animal Science Academy, Chengdu, 610066, China; c State Key Laboratory of Swine and Poultry Breeding Industry, College of Animal Science and Technology, Sichuan Agricultural University, Chengdu, 611130, China; d Jinxin Research Institute for Reproductive Medicine and Genetics, Chengdu Xi Nan Gynecological Hospital Co., Ltd., 66 Bisheng Road, Chengdu, 610000, China

**Keywords:** Primary myoblast, *ST6GAL2*, Cell proliferation and apoptosis, Induced differentiation, Knockout mice, Skeletal muscle

## Abstract

ST6GAL2, a member of the sialoglycosyltransferase family, primarily localizes within the cellular Golgi apparatus. However, the role of the *ST6GAL2* gene in skeletal muscle growth and development remains elusive. In this study, the impact of the *ST6GAL2* gene on the proliferation, differentiation, and apoptosis of primary chicken myoblasts at the cellular level was investigated. Quantitative fluorescent PCR was used to measure the expression levels of genes. Subsequently, using gene knockout mice, we assessed its effects on skeletal muscle growth and development *in vivo*. Our findings reveal that the *ST6GAL2* gene promotes the expression of cell cycle and proliferation-related genes, including *CCNB2* and *PCNA*, and apoptosis-related genes, such as *Fas* and *Caspase-9*. At the individual level, double knockout of *ST6GAL2* inhibited the formation of both fast and slow muscle fibers in the quadriceps, extensor digitorum longus, and tibial anterior muscle, while promoting their formation in the gastrocnemius and soleus. These results collectively demonstrate that the *ST6GAL2* gene facilitates the proliferation, apoptosis, and fusion processes of primary chicken myoblasts. Additionally, it promotes the enlargement of cross-sectional muscle fiber areas and regulates the formation of fast and slow muscle fibers at the individual level, albeit inhibiting muscle fusion. This study provides valuable insights into the role of the *ST6GAL2* gene in promoting proliferation of skeletal muscle.

## 1 Introduction

Livestock farming, with a particular emphasis on domestic chicken farming, holds significant socio-economic importance for individuals residing in low-income nations across Africa and Asia [1]. Domestic chickens are a globally prevalent avian species, largely due to their brief generational span and remarkable adaptability across diverse agroecological settings [2,3]. Domestic chickens are a crucial source of high-quality protein and income for impoverished rural households, making them the most extensively raised livestock species globally [4]. This is due to the presence of the valuable traits of chickens like disease resistance, adaptation to harsh environments, and ability to utilize poor quality feeds [5].

*ST6GAL2*, a member of the sialoglycosyltransferase family, predominantly resides in the Golgi apparatus within cells. Its structural composition encompasses a short N-terminal cytoplasmic tail, a transmembrane domain, a stem domain, and a catalytic domain [6]. Sialic acid has been established as a pivotal contributor to the functional maintenance and structural integrity of skeletal muscle [7,8]. Sialoglycosyltransferases mediate the transfer of β-glycoside donor cytidine 5′-monophosphoric acid-N-acetylneuraminic acid (CMP-Neu5Ac) to the terminal non-reducing positions of glycoprotein and glycoliposaccharide oligosaccharide chains [9,10].

While prior investigations have largely centered on *ST6GAL2* within the context of the brain and tumors, a study on adult brains revealed distinctive tissue-specific patterns of *ST6GAL2* expression, hinting at its potential involvement in specific brain functions [11]. This investigation identified NF-κB and NRSF as transcriptional inhibitors of *ST6GAL2*, whereas neuronal-associated developmental factors Sox5, Purα, and Olf1 were identified as transcriptional activators of *ST6GAL2* [12]. Furthermore, selective knockout of the *dnmt3b* gene in hippocampal excitatory neurons led to compensatory upregulation of *ST6GAL2* expression, impairing object position recognition memory in knockout mice. Transcriptome studies of brain regions in Alzheimer's patients have also unveiled significant downregulation of *ST6GAL2* gene expression, underscoring its potential importance in brain function.

In the realm of cancer, *ST6GAL2* is overexpressed in certain types, including breast cancer, where its elevated expression correlates with poor patient prognosis [13]. Notably, silencing of *ST6GAL2* in follicular thyroid carcinoma has reduced tumor growth *in vivo* models [14]. Additionally, *ST6GAL2* overexpression has been shown to inhibit the Hippo signaling pathway, a tumor suppressor pathway that regulates cell differentiation and proliferation by restraining the YAP and TAZ transcriptional coactivators [15,16].

In contrast to the extensive research on *ST6GAL2* in other contexts, its function in skeletal muscle remains largely unexplored. Sialic acid is crucial in preserving glycoproteins associated with fibrous structure, neuromuscular connectivity, development and regeneration, muscle excitability, and athletic performance in skeletal muscle [17,18]. Despite sialylation being less abundant in muscles than other tissues, muscle tissue is particularly sensitive to mutation-induced sialic acid deficiency, resulting in various diseases, often characterized by a significant loss of exercise capacity [19]. As a sialic acid acyltransferase, *ST6GAL2* may be implicated in physiological processes influenced by sialic acid. Previous studies have indicated limited expression of *ST6GAL2* in adult mouse skeletal muscle and C2C12 myotubular cells, both in tissues and cells [20].

Skeletal muscles constitute roughly 35 % of an individual's total body mass and play a crucial role in facilitating various bodily movements and maintaining proper posture [21,22]. The formation of skeletal muscle is a complex series of events that involves genes responsible for various aspects of muscle development [23]. Skeletal muscle formation is a complex process that ensues after the termination of the cell cycle, guided by an array of regulatory transcription factors such as *MyoD1*, *MyoG*, Myosin heavy chain (*MyHC*), and *Myomaker*. This process involves initiating muscle-specific gene transcription, cell elongation, and cell-to-cell fusion. *MyoD1*, in collaboration with *MyoG*, orchestrates myoblast fusion into multinucleated myotubes, albeit with the distinction that *MyoG* is expressed exclusively during myoblast differentiation, while *MyoD1* is expressed during the skeletal muscle satellite cell period. *MyHC* is a marker for late skeletal muscle differentiation, with elevated levels predicting increased myofiber formation [24]. *Myomaker*, a myoblast membrane fusion protein, is pivotal in activating myoblasts' fusion capacity and contributes significantly to muscle regeneration and formation [25]. The potential impact of *ST6GAL2* knockout on these critical genes remains unclear.

To unravel the role of *ST6GAL2* in skeletal muscle growth and development, this study employs a dual approach. Firstly, it investigates the effects of *ST6GAL2* on myogenesis, differentiation, cell cycle regulation, apoptosis, and proliferation in chicken primary myoblasts *in vitro*, serving as a cellular model. Subsequently, gene knockout mice are generated to explore the *in vivo* effects of *ST6GAL2* on myogenesis, differentiation, and the expression of genes associated with muscle fiber types. The objective of this research is to investigate the function of *ST6GAL2* in skeletal muscle growth, which, to the best of our knowledge, has not been explored before. This study represents the first attempt to explore the impact of ST6GAL2 knockout on skeletal muscle development, thereby contributing original insights to the field and offering valuable insights that can potentially enhance meat yield and quality in livestock production.

## 2 Materials and methods

### 2.1 Experimental materials

Primary myoblasts were isolated using established protocols [26] from 100 Qingjiao chicken eggs from the farm of DEKON GROUP (DEKON, Chengdu, China). The incubation conditions maintained a temperature of 37.8 °C and 60 % humidity. C57/BL6J mice were obtained from Chengdu Dashuo Laboratory Animal Co., LTD, while *ST6GAL2* knockout mice were provided by Jiangsu Jiocainyaokang Biotechnology Co., LTD. For experiments requiring wild-type, single-knockout, and double-knockout mice, breeding procedures were employed (Fig. 1A). Stable F1 generation single-knock mice and WT mice were hybridized, and then continuously bred to obtain the heterogeneous single-knock mice (*ST6GAL2*^+/−^) and double-knock mice (*ST6GAL2*^−/−^). Two pairs of primers (F1/R1 and F2/R2) were used to identify mouse genotypes. Among them, F1/R1 confirmed the presence of double-knock alleles by PCR, and F2/R2 confirmed the presence of wild-type alleles. The double-knock and wild-type bands were 314 bp and 455 bp, respectively (Fig. 1B). Additionally, PCR products were further sequenced to confirm the genotype of the target mouse (Fig. 1C).

**Fig. 1:**
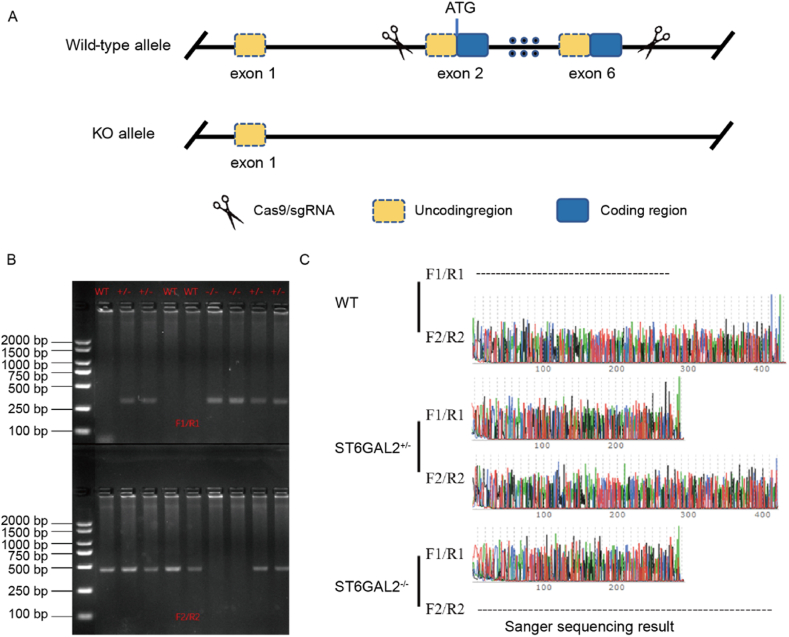
Design and identification of *ST6GAL2* knockout mice. (A) The schematic diagram of the gene knockout; (B) The glue map for genotype identification. The full, non-adjusted images were provided as supplementary material (Figs. S1 and S2). (C) The sequencing results of the PCR products.

### 2.2 Cell culture

Primary myoblasts were isolated from the leg muscles of chicken embryos on the 10th day of incubation. After removing the bones, the leg muscles were placed in a DMEM medium supplemented with 10 % FBS and 0.2 % penicillin/streptomycin. The muscle tissue was then diced and transferred to a 50 ml centrifuge tube. The suspension was agitated for 1 min and passed through a 70 μm cell strainer. The filtrate was centrifugation at 1000 rpm for 5 min at room temperature. The collected cells were resuspended in a growth medium and transferred to culture bottles. The cells were cultured in a 5 % CO_2_ environment at 37 °C and subjected to differential adhesion, a process repeated three times. Cell growth was observed and recorded daily [27].

### 2.3 DNA extraction, RNA extraction, cDNA synthesis and quantitative real-time fluorescence quantitative PCR (qRT-PCR)

DNA was extracted from the toes of one-week-old mice using Tiangen Biochemical Technology Co., LTD. (Blood/cell/tissue genomic DNA extraction kit - centrifugal column DP304) for genotype identification. The DNA concentration was determined using a spectrophotometer. PCR assays were conducted using 2 × Rapid Taq Master Mix (Vazyme-*P*222), and mouse genotypes were identified through electrophoresis on a 1 % agarose gel. RNA extraction from tissues and cells was performed using Trizol, with RNA concentration determined using a spectrophotometer. The synthesis of cDNA chains was accomplished using the ExonScript RT SuperMix with dsDNase reverse transcription kit from the Chengdu Rongwei gene company. Quantification of selected genes was carried out using the LightCycler® Instrument system, employing the chick or mouse β-actin gene as the internal reference. qRT-PCR was performed using the Fast SYBR Green qPCR Master Mix UDG kit from Chengdu Rongwei Gene Co., LTD. All experiments were conducted in triplicate. Relative gene quantification utilized the 2^−ΔΔCt^ method. Primers required for the experiments were synthesized by Qingke Biotechnology Co., LTD., with the primer sequences detailed in Table 1.

**Table 1:** qRT-PCR primers of each detected genes.

Gene	Primer sequence (5′-3′)	Product length (bp)	Tm (°C)	Reference
*Gal-ST6GAL2*	F:TGGAGCCGTCAGAGGAGAATGAG	85	60	XM_046906978.1
	R:GCCCATCCCAGTCATCAGATTCATC			
*Gal-MyoD1*	F:GCTACTACACGGAATCACCAAAT	200	60	[21]
	R:CTGGGCTCCACTGTCACTCA			
*Gal-MyoG*	F:CGGAGGCTGAAGAAGGTGAA	320	60	
	R:CGGTCCTCTGCCTGGTCAT			
*Gal-MyHC*	F:CTCCTCACGCTTTGGTAA	213	60	
	R:TGATAGTCGTATGGGTTGGT			
*Gal-β-actin*	F:TTGTTGACAATGGCTCCGGT	110	58	
	R:AACCATCACACCCTGATGTCT			
*Gal-Myomarker*	F:TGGGTGTCCCTGATGGC	135	51	
	R:CCCGATGGGTCCTGAGTAG			
*Gal-Fas*	F:TCCACCTGCTCCTCGTCATT	78	60	[28]
	R:GTGCAGTGTGTGTGGGAACT			
*Gal-CCNB2*	F:CCTCTTCCACTTCACTTCT	195	51	[29]
	R:CTTTGTACCCCACTTATCA			
*Gal-CCND1*	F:CAGAAGTGCGAAGAGGAAGT	188	51	
	R:CTGATGGAGTTGTCGGTGTA			
*Gal-PCNA*	F:AGCACCAAATCAGGAAAAG	177	51	
	R:GCACAGGAGATGACAACAG			
*Gal-Caspase 3*	F:GGCTACTACTCCTGGAGGA	193	51	
	R:ACACAATGCATGGAATCTG			
*Gal-Caspase 9*	F:GGAGGAGAACAAAAGGACC	187	51	
	R:CTGGAAAAGTTGAATAGGA			
*Mus-MyoD1*	F:CGAGCACTACAGTTGGCGACTAAGAT	204	60	[21]
	R:GCTCCACTATGCTGGACAGGCAGT			
*Mus-MyoG*	F:CCATCCAGTACATTGAGCGCCTACA	241	60	
	R:ACGATGGACGTAAGGGAGTGCAGAT			
*Mus-MyHC*	F:CAAGTCATCGGTGTTTGTGG	158	60	
	R:TGTCGTACTTGGGCGGGTTC			
*Mus-Myomarker*	F:ATCGCTACCAAGAGGCGTT	107	60	
	R:CACAGCACAGACAAACCAGG			
*Mus-MyHC I/Myh7*	F:ACTGTCAACACTAAGAGGGTCA	114	60	[30]
	R:TTGGATGATTTGATCTTCCAGGG			
*Mus-MyHC IIa/Myh2*	F:AAGTGACTGTGAAAACAGAAGCA	222	60	
	R:GCAGCCATTTGTAAGGGTTGAC			
*Mus-MyHC IIb/MYH4*	F:TTGAAAAGACGAAGCAGCGAC	190	60	
	R:AGAGAGCGGGACTCCTTCTG			
*Mus-MyHC IIx/MyHC*	F:GCGAATCGAGGCTCAGAACAA	138	60	[31]
	R:GTAGTTCCGCCTTCGGTCTTG			
*Mus-Tnni1*	F:TGAAGCCAAATGCCTCCACAACAC	155	60	[32]
	R:ACACCTTGTGCTTAGAGCCCAGTA			
*Mus-Tnnc1*	F:AGCTCATGAAGGACGGTGACAAGA	102	61	
	R:AACCGTGCAAGACCAGCATCTACT			
*Mus-Tnni2*	F:AGCAGCAAGGAGCTGGAAGA	101	58	
	R:ATGGCGTCGGCAGACATAC			
*Mus-Tnnc2*	F:CCATCATCGAGGAGGTGGAC	101	60	
	R:CTTCCCCTTCGCATCCTCTT			

Notes: *Gal*: *Gallus gallus domesticus*; *Mus*: *Mus musculus*.

### 2.4 Overexpression and interference efficiency assays

Two overexpression vectors for *ST6GAL2* transcripts (PEGFP-ST-201 and PEGFP-ST-202) were constructed by Nanjing Qingke Biological Co., LTD. Additionally, three si-RNA sequences for gene interference tests were designed and synthesized by Shanghai Sangon Bioengineering Co., LTD., with specific details provided in Table 2.

**Table 2:** *ST6GAL2* (XM_046906978.1) gene interference sequences and control sequences.

Sequence Name	Sequence (5′-3′)	Sequence length (bp)
siRNA-452	GAUUUGGAAGAUGCCUUUAGATT	23
	UCUAAAGGCAUCUUCCAAAUCTT	23
siRNA-862	GCGGUUCAAAGGGAAACGAAATT	23
	UUUCGUUUCCCUUUGAACCGCTT	23
siRNA-1495	GUGUAACGAAGUGCACGUGUATT	23
	UACACGUGCACUUCGUUACACTT	23
Negative Control	UUCUCCGAACGUGUCACGUTT	21
	ACGUGACACGUUCGGAGAATT	21

When cell cultures in T175 flasks reached approximately 80 % confluence, they were trypsinized, counted, and plated. Transfection with overexpression and interference vectors occurred when cell confluence reached 40 %–50 %. The culture medium was replaced 8 h post-transfection. RNA samples were collected for quantitative analysis at 12 and 24 h after transfection to determine transfection efficiency.

### 2.5 CCK-8 assays

Myoblasts in the logarithmic growth phase were harvested, and their cell concentrations were calculated and adjusted accordingly. These cells were then seeded into 96-well plates at a volume of 100 μL per well and cultured in a CO2 incubator. When the cell confluence reached between 40 % and 50 %, transfection was performed using various vectors, including PEGFP-ST-201, PEGFP-N1, Si-NC, and Si-862. At 12, 24, 36, and 48 h post-transfection, 10 μL of CCK-8 reagent was added to each well. The plates were then incubated in the CO2 incubator for an additional 1.5 h. After incubation, the 96-well plate was removed, and the absorbance at 450 nm was measured using a microplate reader under dark conditions to assess cell viability and proliferation.

### 2.6 Cell cycle and apoptosis analysis

Flow cytometry was employed to analyze cell cycle and apoptosis. Cells from the experimental and control groups were collected 24 h after transfection. For cell cycle analysis, samples were washed with PBS and fixed with 70 % ethanol. DNA was stained with propidium iodide (PI) staining solution for 15 min at room temperature. Apoptotic samples were resuspended in 1 × binding buffer and stained in the dark with 5 μL of Annexin V-FITC staining fluorescent dye for 10 min, followed by adding 10 μL of PI for 5 min. Data analysis was conducted using Cytoflex flow cytometry (Beckman Coulter) and Modfit LT (v5.0).

### 2.7 Immunofluorescence

The purity of myoblasts, which are precursor cells that can differentiate into muscle cells, was determined using a technique called desmin immunofluorescence. In this method, a desmin antibody was used to specifically bind to desmin proteins, which are intermediate filament proteins found in muscle cells. To visualize this binding, a secondary antibody, rabbit IgG, conjugated with a fluorescent dye called FITC (Fluorescein isothiocyanate), was employed. This secondary antibody binds to the primary desmin antibody, and the FITC dye allows the desmin protein to be seen under a fluorescence microscope. Cell differentiation was assessed using MYHC and FITC-conjugated anti-mouse IgG antibodies. Nuclear staining was carried out using DAPI (4′,6-diamidino-2-phenylindole), a fluorescent stain that binds strongly to DNA. This allowed the nuclei of the cells to be clearly visualized. Finally, all images were acquired using a fluorescence microscope, which uses specific wavelengths of light to excite the fluorescent dyes (FITC and DAPI), causing them to emit light at different wavelengths. This emitted light is then captured to create detailed images of the cells, highlighting the presence of desmin, MYHC, and the nuclei.

### 2.8 Body weight analysis and growth curve determination

Weekly measurements of mouse body weight were carried out from birth until the 8th week. Each week, the body weight of each mouse was recorded meticulously. The collected body weight data were then categorized into three distinct groups based on the genotype identification results: wild type (WT), heterozygous (ST6GAL2+/−), and homozygous (ST6GAL2−/−). The body weight data for each group were then subjected to statistical analysis using GraphPad Prism 9, a software tool commonly used for scientific graphing and data analysis. The software was used to generate detailed growth curves for the mice, illustrating the changes in body weight over time for each genotype group. This analysis provided insights into the growth patterns and potential differences among the wild-type, heterozygous, and homozygous mice.

### 2.9 Hematoxylin-eosin (HE) stain

Paraffin-embedded tissue sections were first deparaffinized and then oven-dried at 65 °C to prepare them for staining. The sections were then stained with hematoxylin for 2 min to highlight the cell nuclei. Following the hematoxylin staining, the sections were rinsed thoroughly with tap water for 5 min to remove excess stain. Next, the cells underwent differentiation for 15–20 s to enhance the contrast between the nuclei and the cytoplasm, followed by another rinse with tap water for 3 min. After differentiation, the sections were stained with eosin for 40 s to stain the cytoplasm and other tissue components. This was followed by a final rinse with tap water for 3 min to ensure all excess eosin was removed. Once the staining process was complete, the sections were dehydrated through a series of alcohol washes to remove any remaining water. The dehydrated sections were then mounted using neutral resin to preserve the tissue and facilitate microscopic examination. Observations and imaging of the stained sections were performed using a slide scanner to capture detailed images for further analysis.

### 2.10 Statistical analysis

Throughout this study, Various software tools were utilized, including Primer 5.0 for primer design, BioRad CFX for real-time PCR data analysis, SPSS 8.0 for statistical analysis, GraphPad Prism 9 for graph plotting, and Image Plus (v1.4) for image analysis. For the analysis of experimental data from quantitative PCR, the 2^-(ΔΔCt)^ method was employed [33], and GraphPad Prism 9.0 was used to plot the data and generate graphs. Results were presented as Mean ± standard error (SEM) [34]. Significance levels were determined on *P*-values: results were considered non-significant when *P* > 0.05; significant when 0.01 < *P* < 0.05, indicated by a single asterisk (“*"); and extremely significant when *P* < 0.01, indicated by double asterisks (“**"). Each dataset included at least three biological replicates to ensure the reliability and reproducibility of the results.

## 3 Results

### 3.1 Identification of chicken primary myoblasts

**Fig. 2:**
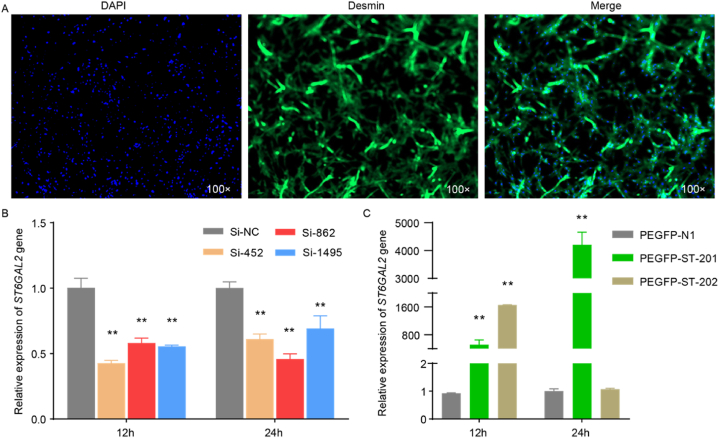
Identification of chicken primary myoblasts by immunofluorescence, overexpression, and interference efficiency of myoblasts. **(A)** Identification of chicken primary myoblasts by immunofluorescence. **(B)** Interference efficiency of the *ST6GAL2* gene at 12 h, 24 h, and 48 h; **(C)** Overexpression efficiency of the *ST6GAL2* gene at 12 h and 24 h. Note: The data above were presented as mean ± SEM (n = 3), error bars show the SEM of the triplicate; (*) represents significant (*P* < 0.05), (**) represents extremely significant (*P* < 0.01).

To verify the purity of the primary myoblasts, we conducted immunofluorescence to detect the cell-specific expression of desmin protein. The results demonstrated that most F1 generation cells were myoblasts after 24 h of culture, confirming their suitability for further study (Fig. 2A).

To modulate the mRNA expression of the *ST6GAL2* gene in chicken myoblasts, we designed and synthesized *ST6GAL2* interfering Si-RNA (Si-452, Si-862, Si-1495) and eukaryotic overexpression vectors (PEGFP-ST-201, PEGFP-ST-202). These constructs were then transfected into primary myoblasts from chickens. Cells were collected at 12 h, 24 h, and 48 h post-transfection, and the fluorescence quantitative detection of the target gene *ST6GAL2* and the internal reference gene *β-actin* was performed to analyze interference efficiency and overexpression efficiency. The results revealed that the expression of *ST6GAL2* in primary myoblasts transfected with interfering RNA exhibited a significant downregulation at 12 h and 24 h (*P* < 0.01) compared to the control group (Fig. 2B). Si-862 displayed stable interference efficiency at both time points, with the interference effect gradually increasing. In the overexpression test, the relative expression of *ST6GAL2* mRNA significantly increased (*P* < 0.001) at both 12 h and 24 h post-PEGFP-ST-201 transfection, particularly at 24 h, where it exceeded 1000-fold, confirming the successful construction of the overexpression vector. The overexpression effect of PEGFP-ST-202 diminished significantly at 24 h (Fig. 2C). Based on these results, Si-862 and PEGFP-ST-201 were selected for subsequent interference and overexpression tests, with 24 h identified as the optimal transfection time point.

### 3.2 ST6GAL2 promotes the proliferation and apoptosis of primary chicken myoblasts

**Fig. 3:**
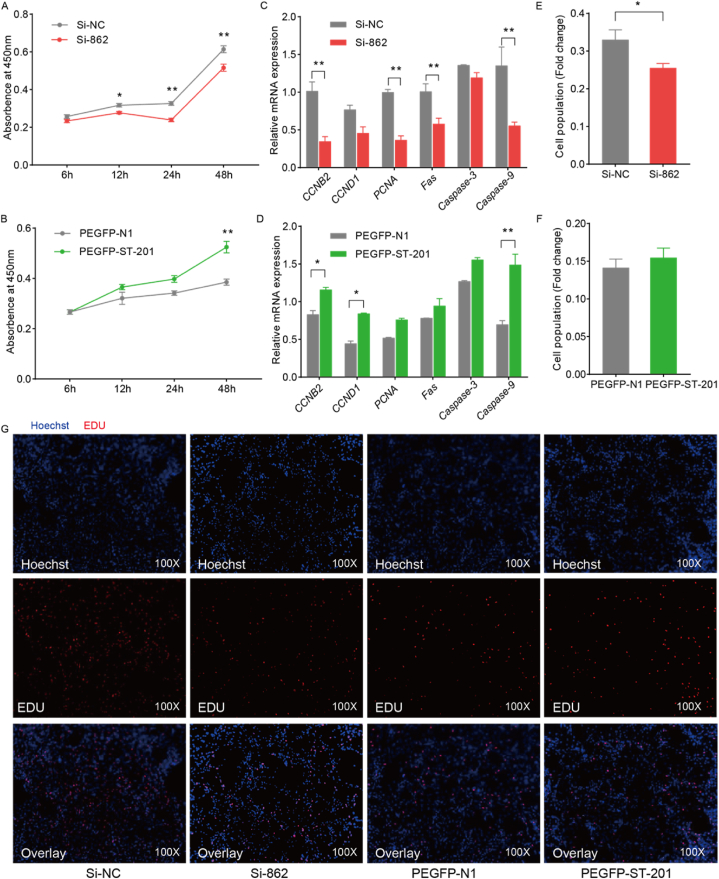
Effect of *ST6GAL2* on myoblast proliferation. Light absorption values of chicken primary myoblasts at 450 nm after interference and overexpression with *ST6GAL2* using Si-862 **(A)** and PEGFP-ST-201 **(B)**, respectively. Detection of cell apoptosis-related marker gene expression after interference **(C)** and overexpression **(D)** with the *ST6GAL2* gene. From left to right on the horizontal axis: cyclin B2 (*CCNB2*), cyclin 1 (*CCND1*), proliferating cell nuclear antigen (*PCNA*), cell indicative death receptor (*Fas*), *Caspase-3*, *Caspase-9*. The influence of the *ST6GAL2* gene interference **(E)** and overexpression **(F)** after EdU detection on myoblast proliferation. **(G)** Microscopic results of the EdU experiment after transfection of Si-862 and PEGFP-ST-201.

Chicken primary myoblasts were cultured in 96-well cell plates, and when the cell density reached 25 %, they were transfected according to the grouping with PEGFP-N1, PEGFP-ST-201, Si-NC, and Si-862. The mass of transfected plasmid per well in the PEGFP-N1 and PEGFP-ST-201 groups was 0.15 μg, while the concentration of Si-NC and Si-862 transfected per well was 10 nM, with 6 replicates per group. CCK8 results indicated that interference with *ST6GAL2* gene led to significant inhibition of chicken myoblast viability at 12 h (*P* < 0.05), followed by a notable increase in cell viability compared to the control group at 24 h and 48 h (Fig. 3A). There was no significant difference in cell viability between overexpression *ST6GAL2* and the control group before 24 h, but at 48 h, the viability of chicken myoblasts was significantly and progressively enhanced (*P* < 0.01) (Fig. 3B). These findings suggest that *ST6GAL2* may play a role in promoting the proliferation of chicken primary myoblasts.

To investigate how the *ST6GAL2* gene influences the proliferation process of chicken myoblasts, we examined the expression changes of cell cycle and apoptosis-related genes upon *ST6GAL2* gene overexpression and interference via qRT-PCR. Primary myoblasts from chickens were transfected with PEGFP-N1, PEGFP-ST-201, Si-NC, and Si-862 groups when the cell density reached 40–50 %. PEGFP-N1 and PEGFP-ST-201 plasmids were transfected at 4 μg per well, while Si-NC and Si-862 were transfected at 80 nM per well. The qRT-PCR results revealed that upon interference with the *ST6GAL2* gene, the expression of cell cycle and proliferation-related genes *CCNB2* and *PCNA* was significantly downregulated (*P* < 0.01), whereas the expression of *CCND1* was not significantly affected (*P* > 0.05). Additionally, the expression of apoptosis-related genes *Fas* and *Caspase-9* was markedly reduced (*P* < 0.01), while *Caspase-3* expression remained unchanged (*P* > 0.05) (Fig. 3C). Similarly, overexpression of the *ST6GAL2* gene resulted in a significant upregulation of *CCNB2* and *CCND1* expression (0.01 < *P* < 0.05), with no significant change in *PCNA* expression. However, among apoptosis-related genes, only *Caspase-9* showed a significant increase (*P* < 0.01), while the other two genes exhibited no significant changes (Fig. 3D).

We further employed the EdU assay to assess the impact of *ST6GAL2* on the proliferation of chicken myoblasts. The results indicated that disturbance of *ST6GAL2* gene expression led to a significant reduction in the proliferation of chicken primary myoblasts (0.01 < *P* < 0.05) (Fig. 3E). Conversely, overexpression of the *ST6GAL2* gene appeared to promote chicken primary myoblast proliferation, although this effect was not statistically significant (*P* > 0.05) (Fig. 3F and G). Thus, the *ST6GAL2* gene appears to have a promoting effect on chicken myoblast proliferation.

**Fig. 4:**
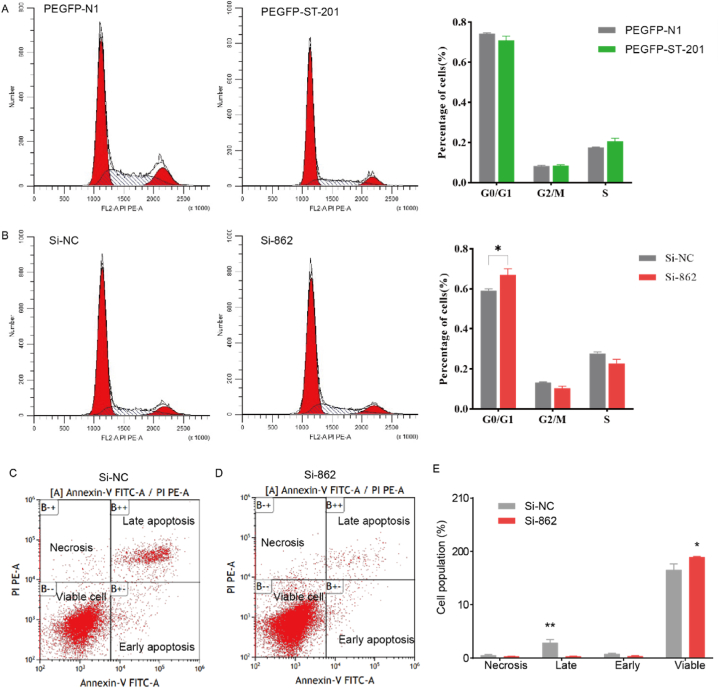
Effect of the *ST6GAL2* gene on apoptosis of myoblasts. Cell cycle detection results by flow cytometry of *ST6GAL2* overexpression **(A)** and *ST6GAL2* interference **(B)**. Flow cytometry results are shown on the left and quantified flow cytometry results are shown on the right. (**C)** and **(D)** represent scatter plot results of apoptosis after Si-NC and Si-862 transfection, respectively. **(E)** The statistical results of flow cytometry after transfection of Si-862.

To gain further insights into the role of the *ST6GAL2* gene in the apoptosis process of chicken myoblasts, we investigated the cycle distribution of myoblasts in each group 24 h after transfection PEGFP-N1, PEGFP-ST-201, Si-NC, and Si-862 using flow cytometry. Compared with the control group (PEGFP-N1), the number of primary myoblasts in the G0/G1 phase decreased to a certain extent after overexpression of *ST6GAL2*, while the number of the S phase increased. These effects were not significant (*P* > 0.05) (Fig. 4A). Additionally, after interfering with the *ST6GAL2* gene, the number of primary myoblasts in the G1 phase increased significantly (*P* < 0.05) (Fig. 4B), while the number of cells in the G2 and S phases decreased, indicating that the cells were blocked in the G1 phase, the DNA synthesis and DNA replication of the cells were relatively reduced. These results demonstrated that interference had a more significant effect on cell proliferation than the overexpression of *ST6GAL2*. Similarly, we also employed flow cytometry to investigate the effect of *ST6GAL2* gene interference on myoblastic apoptosis. The results revealed that interference with *ST6GAL2* gene expression significantly decreased the rate of late apoptosis in chicken myoblasts (*P* < 0.01) and increased cell viability (*P* < 0.01) (Fig. 4C–E).

### 3.3 ST6GAL2 gene promotes expression of myomaker in chicken primary myoblast

**Fig. 5:**
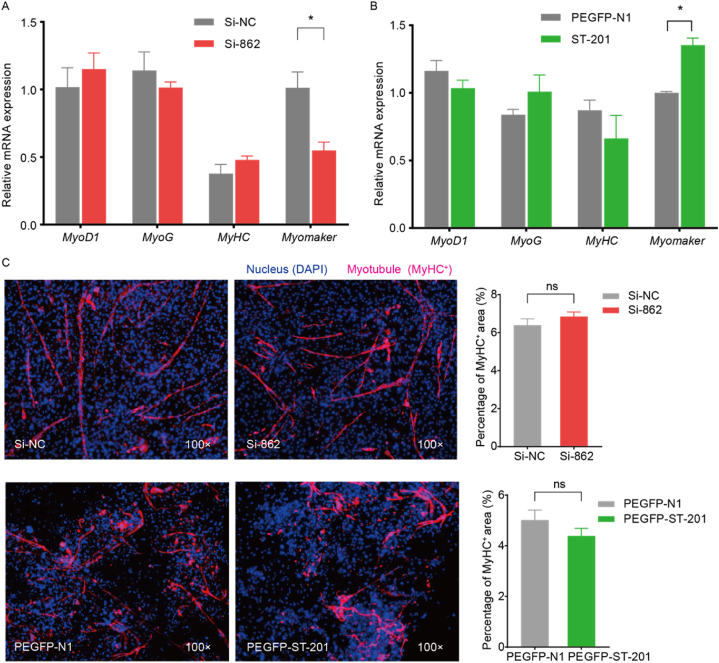
Expression of myoblast-related marker genes after interference (A) and overexpression (B) with *ST6GAL2* gene (n = 3). Myoblast regulatory factor (*MyoD1*), myogenic hormone (*MyoG*), myosin heavy chain (*MyHC*), and Myoblast fusion factor (*Myomaker*). (C) *MyHC* staining of chicken myoblasts after 72 h of interference and overexpression with *ST6GAL2*. Note: *MyHC* positive in myotubule (red), DAPI positive in the nucleus (blue); right panels show *MyHC* positive area statistics (n = 6). (For interpretation of the references to colour in this figure legend, the reader is referred to the Web version of this article.)

To investigate the role of the *ST6GAL2* gene in the differentiation and fusion of chicken primary myoblasts, we cultured these myoblasts in 6-well cell plates and transfected them with PEGFP-N1, PEGFP-ST-201, Si-NC, and Si-862 when they reached a density of 50 %. Upon reaching 80–90 % confluence, the culture medium was replaced with a differentiation medium. RNA samples were collected 72 h after induction of differentiation for qRT-PCR analysis. After Si-862 transfection, it was observed that the expression of *MYOD1*, *MYOG*, and *MyHC* genes did not change (Fig. 5A). However, the expression of *Myomaker* was significantly downregulated (*P* < 0.05) compared to the Si-NC group (Fig. 5A), Furthermore, the expression of *Myomaker* was notably increased following PEGFP-ST-201 transfection (*P* < 0.05) (Fig. 5B). Consistently, *MyHC* immunofluorescence results indicated no significant difference in the *MyHC*-positive area after *ST6GAL2* overexpression and interference compared to the control group (Fig. 5C).

### 3.4 ST6GAL2 absence affects adult mice skeletal muscle characteristics of gene expression and morphology

**Fig. 6:**
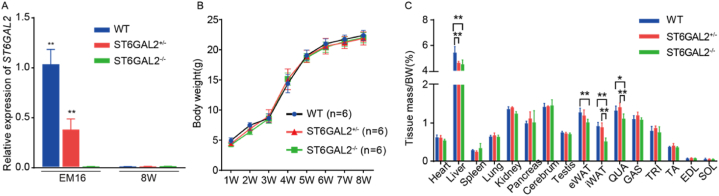
*ST6GAL2* absence affects adult mice skeletal muscle characteristics of gene expression and morphology. (A) Expression of *ST6GAL2* in mouse muscles at Day 16 of the embryonic stage (EM16) and 8 weeks after birth (8W). (B) Body weight growth curves of mice with different genotypes. (C) Tissue weight analysis of mice with different genotypes at 8 weeks of age.

To delve further into the function of *ST6GAL2*, we employed *ST6GAL2* knockout mice to explore its impact on muscle development. We targeted exon 2 to exon 6 of the *ST6GAL2*-201 (ENSMUST00000025000.3) transcript for knockout using CRISPR/Cas9 technology, as completed by Jiangsu Jicui Yaokang Biological Technology Co., Ltd. The knockout strategy is illustrated in Fig. 1. Expression of *ST6GAL2* in chicken skeletal muscle showed higher expression on day 9 of embryonic incubation (Carnegie stages 22–23). In alignment with the corresponding embryonic periods, we examined the expression of *ST6GAL2* at day 16 (Carnegie stage 23) of mouse embryonic development. The expression of *ST6GAL2* in muscle tissue was quantitatively analyzed at two-time points. The results revealed that *ST6GAL2* was nearly absent in adult mouse skeletal muscle (Fig. 6A).

As comprehensive observation of the specific effects of *ST6GAL2* on various types of skeletal muscle in mice during the embryonic stage was challenging, we selected 8-week-old (adult) mice to investigate the function of *ST6GAL2* in skeletal muscle growth and development. During the first week, wild-type mice exhibited significantly higher body weight than double-knockout mice (*P* < 0.05). However, there were no significant differences in body weight (*P* > 0.05) during the subsequent six-time points at 3, 4, 5, 6, 7, and 8 weeks (Fig. 6B). These results suggest that *ST6GAL2* knockout primarily plays a role in early development. Further analysis of the collected weight of 16 tissues indicated that both *ST6GAL2* ± and *ST6GAL2*−/− mice exhibited significantly reduced liver and groin fat weights (*P* < 0.01) compared to wild-type mice. Moreover, *ST6GAL2*−/− mice displayed a significant reduction in gonadal fat weight (*P* < 0.01) compared to wild-type mice. In the target muscle tissue, Quadriceps muscle (QUA) weight in *ST6GAL2*−/− mice was significantly decreased (*P* < 0.05) compared to both wild-type and *ST6GAL2* ± mice. However, this difference was not significant in the remaining five muscle sites (*P* > 0.05) (Fig. 6C).

**Fig. 7:**
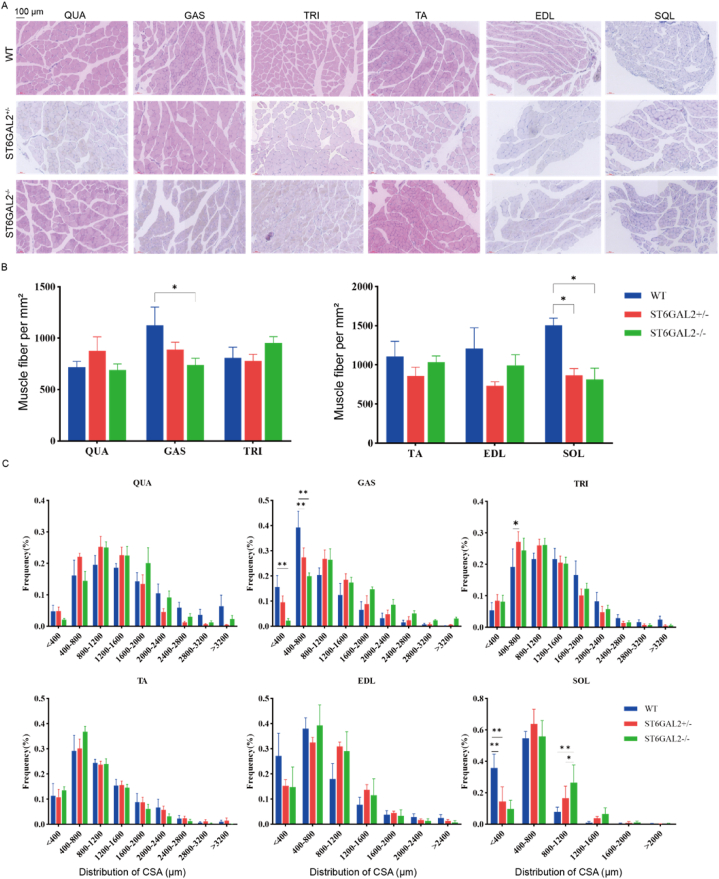
The characteristics and parameters of muscle fibers in different mice muscle parts. **(A)** Histological section of skeletal muscle of six parts. **(B)** The number of myofibers in each skeletal muscle tissue. **(C)** Muscle fiber cross-sectional area (CSA) of each muscle. Quadriceps muscle (QUA), gastrocnemius muscle (GAS), triceps brachii (TRI), tibial anterior muscle (TA), Extensor digitorum longus (EDL), and soleus muscle (SOL).

To assess the effect of *ST6GAL2* knockout on the morphology of muscle fibers in mouse skeletal muscles, we prepared paraffin sections of six muscle parts from 8-week-old mice, stained them with HE (Fig. 7A), and conducted microscopic scanning to observe the morphological characteristics of muscle fibers. Image-Pro Plus 6.0 was used to analyze the cross-sectional area and number of muscle fibers stained by HE in each muscle site. The results demonstrated that both gastrocnemius muscle (GAS) and soleus muscle (SOL) exhibited a decreased number of fibers compared to wild-type mice (Fig. 7B).

Moreover, the cross-sectional area of muscle fibers in the QUA increased within the range of 800–1600 mm after single and complete knockout, while the number of muscle fibers with a larger cross-sectional area decreased to some extent. In the GAS, knockout resulted in a decrease in the number of myofibers within the 0–800 mm range, accompanied by an increase in the number of myofibers with a larger cross-sectional area. Similar trends were observed in the extensor digitorum longus (EDL). In the triceps brachii (TRI), a higher proportion of myofibers exhibited a smaller cross-sectional area (Fig. 7B). However, in the tibial anterior muscle (TA) of the three genotypes of mice, there was no significant change in the distribution of muscle fiber cross-sectional area. Additionally, the number of myofibers smaller than 400 mm was significantly reduced in the SOL (*P* < 0.01), while the number of myofibers with a cross-sectional area of 800–1200 mm increased significantly (*P* < 0.01 or *P* < 0.05) (Fig. 7C). These findings suggest that *ST6GAL2* knockout promotes the enlargement of muscle fibers in the GAS, EDL, and SOL, whereas this effect is less pronounced in other muscle parts.

### 3.5 ST6GAL2 absence affects adult mice muscle gene expression

**Fig. 8:**
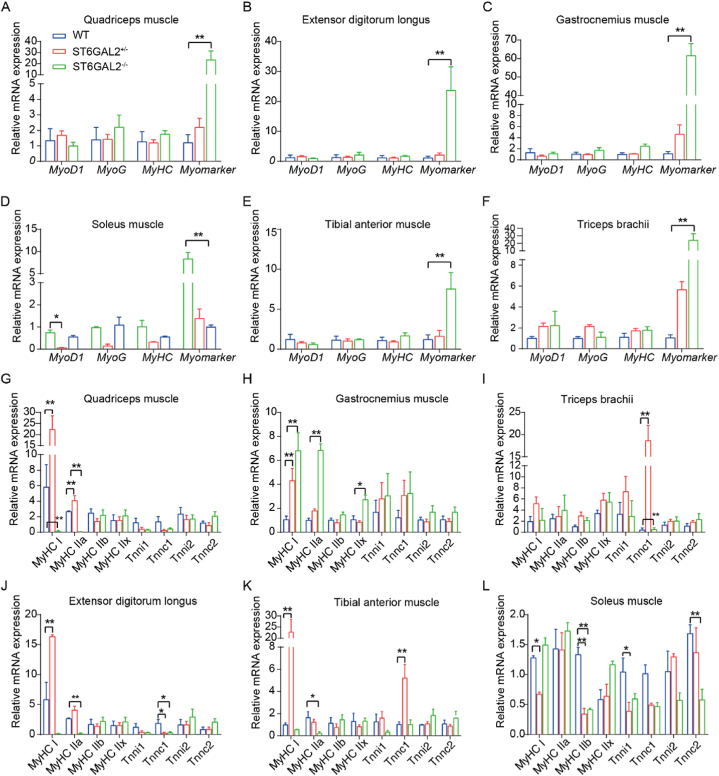
Expression of skeletal muscle development-related genes (A–F), and the fiber type-related genes (G–L) in six muscle tissues of mice.

We proceeded to assess the mRNA levels of *MyoD1*, *MyHC*, *MyoG*, and *Myomaker* in the skeletal muscles of 8-week-old mice with three distinct genotypes. These genes serve as markers associated with muscle proliferation, differentiation, and fusion. The findings showed a slight tendency for increased *Myomaker* expression in the skeletal muscles of single-knock mice compared to wild-type mice, but this trend did not reach statistical significance (*P* > 0.05). Nevertheless, upon complete knockout of the *ST6GAL2* gene, the expression of *Myomaker* in all muscle sites consistently exhibited a significant increase (*P* < 0.01) (Fig. 8A–F). Additionally, the expression of *MyoD1*, *MyHC*, and *MyoG*, related to muscle proliferation and differentiation, did not undergo significant changes in any of the six muscle tissues. These results suggest that *ST6GAL2* may exert an inhibitory effect during muscle fusion.

To further explore the role of *ST6GAL2* in transforming myofiber types, we examined the expression of fast-twitch and slow-twitch muscle genes in the six muscle tissues of mice with three genotypes. Fast-twitch muscle fiber marker genes, including *MyHC IIa*, *MyHC IIb*, and *MyHC IIx*, along with *Tnni2* and *Tnnc2*, were investigated, while slow-twitch fiber marker genes encompassed *MyHC I*, *Tnni1*, and *Tnnc1*. The results demonstrated that complete knockout of *ST6GAL2* led to a significant downregulation of *MyHC I* expression (*P* < 0.01) and *MyHC IIa* expression (*P* < 0.01) (Fig. 8G) in QUA. In the GAS, both single-knock and double knockout significantly increased the expression of *MyHC I* (*P* < 0.01), with the double knockout effect being more pronounced. *MyHC IIa* expression showed an extremely significant increase (*P* < 0.01) in *ST6GAL2*−/− mice (Fig. 8H). *MyHC IIa* expression also increased in *ST6GAL2* ± mice, although the difference was not statistically significant.

Conversely, in the TRI (Fig. 8I) and TA muscle (Fig. 8J), most gene expressions remained largely unchanged. *Tnnc1* expression at both sites was concentrated in single-knock mice and was several times higher than in wild-type and double-knock mice (*P* < 0.01). In addition, in the EDL, a feather-like muscle of the anterior (extensor) compartment of the leg, the expression of both *MyHC I* and *MyHC IIa* in *ST6GAL2* ± mice significantly increased (*P* < 0.01) compared to wild-type mice, while in *ST6GAL2*−/− mice, their expression markedly decreased (*P* < 0.01), showing an inverse trend. The changes of *Tnnc1* in both mice showed a significant decrease in expression (0.01 < *P* < 0.05) (Fig. 8K). In the SOL, the expression of *MyHC IIb* in *ST6GAL2* ± and *ST6GAL2*−/− mice was significantly downregulated (*P* < 0.01). Additionally, compared to WT mice, single-knock significantly downregulated *Tnni1* expression in the SOL (0.01 < *P* < 0.05), while double knockout significantly downregulated the expression of *Tnnc2* (*P* < 0.01) (Fig. 8L).

## 4 Discussion

### 4.1 ST6GAL2 gene promotes the proliferation and apoptosis of chicken primary myoblasts

Myogenesis encompasses various crucial processes, including myoblast proliferation, differentiation, apoptosis, and fusion, with genes regulating these processes providing insights into myogenesis progression. Cyclin D1 (*CCND1*) plays a pivotal role in regulating the G1 phase of the cell cycle, and its activation by cyclin-dependent kinase 4 (CDK4) drives the cell cycle from G1 to S phase [35]. Cyclin B2 (*CCNB2*) serves as a critical cell cycle regulator, being synthesized in the G1 phase and subsequently downregulated, and defects in *CCNB2* have been associated with abnormal cell division and cell cycle arrest [36,37]. Proliferating nuclear antigen (*PCNA*) is a crucial factor in DNA synthesis and cell cycle progression, and its inhibition can impede the cell cycle [38].

In our study, interference with the *ST6GAL2* gene significantly reduced the expression of *CCNB2* and *PCNA*, while overexpression of the gene resulted in a notable increase in *CCNB2* and *CCND1* expression. Which showed the positive regulation effect of *ST6GAL2* on these genes. In addition, the apoptosis is regulated by many pathways and genes, the gene *fas* might not have such significant effect on muscle death here. Additionally, EdU assays indicated that reduced *ST6GAL2* expression inhibited cell proliferation. Flow cytometry analysis further revealed an increase in the number of cells in the G0/G1 phase following *ST6GAL2* gene interference, accompanied by a decrease in the G2 and S phases, implying that more cells were arrested in the G0/G1 phase. Together, these findings demonstrate that the *ST6GAL2* gene promotes the proliferation of chicken primary myoblast.

Apoptosis, a programmed cell death process, involves the activation, expression, and regulation of various genes, including *Caspase-3*, *Fas*, and *Caspase-9* [39,40]. Mammals possess two apoptotic pathways: the extrinsic pathway, which is extrinsic receptor-induced, and the intrinsic mitochondrial stress-induced pathway, which involves cascades of caspase [41]. Both overexpression and interference of *ST6GAL2* in myoblasts resulted in significant changes in *Caspase-9* expression. *Caspase-9* has been shown to participate in the apoptosis process by mediating mitochondrial disruption and increasing the production of reactive oxygen species (ROS) [40]. Furthermore, *Fas* was significantly downregulated in the interference group. *Fas*, a cell surface death receptor, is ubiquitously expressed in cells and can induce apoptosis through interactions with its ligand FasL [42]. Subsequent flow cytometry apoptosis assays demonstrated that *ST6GAL2* interference reduced myoblast apoptosis. These results collectively suggest that *ST6GAL2* gene expression promotes myoblastic apoptosis through both the receptor-induced and mitochondrial-induced apoptotic pathways.

### 4.2 ST6GAL2 gene regulating the balance between myoblast proliferation and differentiation

In the context of myoblast differentiation, the changes in *Myomaker* expression resulting from *ST6GAL2* gene overexpression and interference were not accompanied by alterations in *MyoD1* and *MyoG* gene expression. This suggests that *ST6GAL2* might directly regulate *Myomaker* gene expression or act through other transcription factors. For instance, previous studies have identified miR-140–3p, miR-491, and miR-16-1 as binding to the 3′-UTR of *Myomaker* in both mice and chickens [43,44].

Myoblast fusion is a multi-step process involving two distinct stages mediated by *Myomaker* and *Myomerger*. *Myomaker* facilitates the formation of hemifusions, while *Myomerger* acts on cell membranes to generate membrane stress that enables complete fusion independently of *Myomaker* [45]. In our study, decreased *ST6GAL2* expression can significantly increase the expression of *Myomaker*. *Myomaker* is a muscle-specific membrane protein that regulates myoblast fusion during early embryonic development in mice [46]. In adult mouse skeletal muscle, *Myomaker* is typically expressed at low levels but is reactivated during muscle fiber repair following injury [47,48]. Thus, the significant upregulation of *Myomaker* in adult mice observed in this study also suggests potential muscle fiber formation.

Furthermore, the coordinated regulation of myoblast proliferation, differentiation, and apoptosis is essential for skeletal muscle formation [49]. Previous work by Dehkordi et al. demonstrated that overexpression of *CCND1* inhibits *MyoD1*-induced myogenesis and transcriptional activation [50]. Consistent with these findings, our study revealed that the overexpression of the *ST6GAL2* gene promotes *CCND1* expression, and leads to a decrease in *MyoD1* expression. This suggests that *ST6GAL2* may play a role in modulating the balance of myoblast proliferation and differentiation, possibly through regulating *CCND1* and *MyoD1* expression levels.

### 4.3 ST6GAL2 genes are involved in the regulation of muscle fiber types and size in different types of muscle

Changes in muscle fiber size are a fundamental aspect of muscle plasticity adaptation and are influenced by various factors, including regulating growth and development processes and environmental factors. Typically, alterations in muscle fiber size are critical indicators of muscle hypertrophy and atrophy. Notably, muscle fiber cross-sectional area significantly increases in response to resistance training-induced hypertrophy [51]. Ohno et al. also reported increased myofiber cross-sectional area in lactic acid-induced hypertrophy of the TA muscle [52]. Conversely, muscle fiber cross-sectional area decreases significantly in muscles experiencing atrophy, such as the GAS and TA muscles in mice with muscle atrophy [53,54]. In this study, the knockout of the *ST6GAL2* gene led to an increased muscle fiber cross-sectional area in the GAS, EDL, and SOL. This observation suggests that the *ST6GAL2* gene inhibits the increase in muscle fiber size during the growth and development of specific muscle groups in mice. In addition, after *ST6GAL2* gene was knocked out, not every muscle was affected and the significant effect on each muscle is different, and our results provided preliminary visualization results for this purpose.

The function of skeletal muscle is often achieved through changes in the proportion of different muscle fiber types within the muscle [55]. The diversity and variations in fiber type are primarily attributed to the differential accumulation of specific proteins, which is controlled by the regulation of protein synthesis and degradation. Research has indicated that *MyHC* subtypes are the primary proteins responsible for muscle strength production and constitute approximately 25 % of total muscle protein [56]. *MyHC* subtypes also serve as cell type-specific markers for identifying signaling pathways that govern muscle cell identity [57]. In this study, the expression of *MyHC I* (a marker for oxidative type I slow-twitch muscle fiber) was significantly upregulated in the fast-twitch EDL and hybrid muscles (QUA, GAS, TA) of single-knockout mice. Conversely, its expression was reduced in the slow-twitch SOL muscle, suggesting changes in the ratio of type I muscle fibers in these muscles. Moreover, the expression of *Tnnc1* significantly rose in the TRI and TA of single-knockout mice, suggesting that *Tnni1* enhances the interaction between actin and myosin by binding to calcium [58]. These findings suggest that single-knockout *ST6GAL2* may promote the formation of slow-twitch muscles in the QUA, GAS, TRI, TA, and EDL; while inhibiting slow-twitch muscle formation in the SOL muscle. However, the expression of both *MyHC I* and *MyHC IIa* (type IIa fast-twitch muscle fibers) decreased dramatically after *ST6GAL2* double knockout in QUA, EDL, and TA muscles, suggesting that the loss of *ST6GAL2* function in these muscles led to impaired myofiber formation. In contrast, the increase in both type I slow-twitch and type IIa fast-twitch muscle fibers in GAS and SOL, two adjacent muscles, suggests the involvement of genes other than *ST6GAL2* in these muscles. Further research is needed to understand more complex scenarios.

There are limitations for our study, according to the existing results, *ST6GAL2* gene has no effect on the differentiation of myoblasts. The dysregulation of myomaker expression may be caused by other potential molecular mechanisms, which still need to be further explored. We have provided a preliminary description of the changes observed in multiple muscle tissues of *ST6GAL2* gene-knockout mice, and the type of muscle fibers may be helpful in explaining these changes, although not every part of the muscle had distinct change. These can serve as a targeted research direction for further studies.

## 5 Conclusions

The specific role of *ST6GAL2* in skeletal muscle is not fully understood, as this is a relatively new class of enzymes, and most research on the ST6GAL family of enzymes has focused on the immune system and cancer. Therefore, we first studied the effect of *ST6GAL2* on muscle function by conducting interference and overexpression experiments on primary myocytes, mainly to assess cell proliferation, apoptosis, and *myomaker* expression, and the results showed that: (1) *ST6GAL2* promotes cell proliferation and myocyte fusion process. Then, phenotypic analysis of skeletal muscle in six parts of the *ST6GAL2* knockout mice and detection of muscle-related molecular markers indicated that (2) *ST6GAL2* genes are involved in the changes of fast-twitch and slow-twitch may be the main influencing mode. Therefore, more experimental data and studies may be needed to elucidate the specific mechanisms of *ST6GAL2* in skeletal muscle.

## Funding

This research was supported by the 10.13039/501100012166National Key R & D Program of China (2022YFF1000100 and 2023YFD1300400), the Sichuan Science and Technology Program (2021YFYZ0009 and 2021ZDZX0008), the 10.13039/501100001809National Natural Science Foundation of China (32225046).

## Ethics statement

Animal experiments conducted in this study adhered to the Guidelines for Experimental Animals established by the Ministry of Science and Technology (Beijing, China, revised in March 2017). Ethical approval for the study was obtained from the Ethics Committee of Sichuan Agricultural University (protocol number 2020202010).

## Informed consent statement

Not applicable.

## Data availability statement

The data presented in this study are not publicly available and are available on request from the corresponding author.

## CRediT authorship contribution statement

**Tao Wang:** Writing – original draft, Formal analysis, Data curation. **Bo Ran:** Writing – original draft. **Yingyu Luo:** Data curation. **Jideng Ma:** Formal analysis. **Jing Li:** Formal analysis. **Penghao Li:** Methodology. **Mingzhou Li:** Writing – review & editing, Conceptualization. **Diyan Li:** Writing – review & editing, Visualization, Formal analysis, Conceptualization.

## Declaration of competing interest

The authors declare that they have no known competing financial interests or personal relationships that could have appeared to influence the work reported in this paper.

## Figures and Tables

**figs1:**
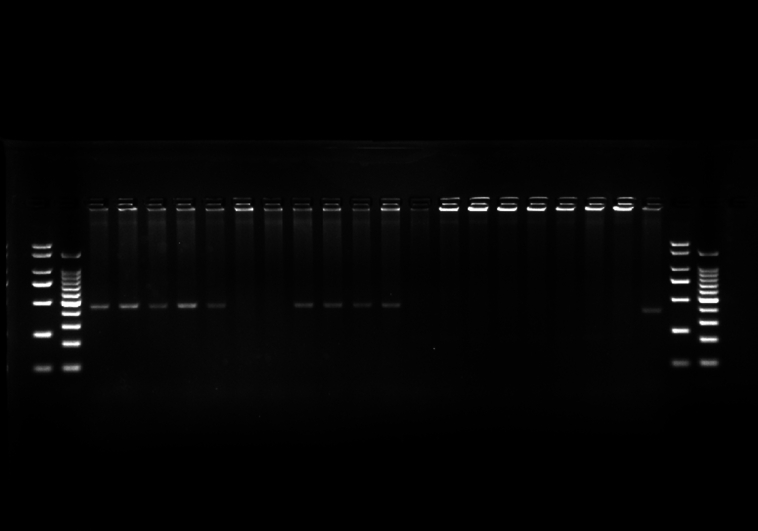


**figs2:**